# Repeated visitations of spermatophores and polyandry in females of eriophyoid mites

**DOI:** 10.1007/s10493-013-9756-9

**Published:** 2013-11-15

**Authors:** Katarzyna Michalska

**Affiliations:** Department of Applied Entomology, Warsaw University of Life Sciences, Nowoursnowska 159, 02-776 Warsaw, Poland

**Keywords:** Polyandry, Eriophyoidea, *Aculops allotrichus*, *Cecidophyopsis hendersoni*, Sex dissociation, Sperm storage

## Abstract

Eriophyoid females store sperm either asymmetrically in one spermatheca, or symmetrically in both spermathecae. Previous studies have suggested that species in which females store sperm asymmetrically pick up sperm from only one spermatophore, while those with symmetrical sperm storage pick up sperm from two or more spermatophores during their lifetime. The aim of this study was to examine spermatophore visitation behaviour and symmetry of sperm storage in *Aculops allotrichus* from the black locust tree and *Cecidophyopsis hendersoni* from the yucca. This would indicate monandry or polyandry in these species. In both eriophyoids, the spermatophore visitation consisted of three phases: mounting, lying on the spermatophore and dismounting. *Aculops allotrichus* stored sperm asymmetrically. However, nearly one-third of the observed females visited two spermatophores, rather than only one in their lives. When *A. allotrichus* females visited two spermatophores they spent a similar amount of time at the first and at the second visitation. Also, the times of visitation of the first of the two spermatophores and the single spermatophore in a female lifetime did not differ significantly. This would suggest that apart from monandry, double insemination also occurs in this species. By contrast, *C. hendersoni* females were polyandrous. They stored sperm symmetrically and visited several spermatophores, on average 1.54 (max 6) per day, and up to 33 spermatophores in their lives. The benefits of repeated spermatophore visitation and the possible mechanisms of sperm storage in both species are discussed.

## Introduction

Considering sexual disparity in gamete investments (anisogamy) males should maximise their reproductive success by mating with many females, while females should choose the highest quality partner and mate only to the extend which will ensure fertilization of all her eggs (Bateman 1948; Trivers 1972). In monandrous insects and mites, sperm obtained from a single insemination can be sufficient to fertilize eggs produced during the whole female life span (Eberhard 1996; Simmons 2001). Monandry may be beneficial, as multiple matings can incur some serious costs to females: e.g. wasted time and energy used for finding a partner and mating, increased exposure to predation, parasites and diseases, harm from aggressive males, etc. (Simmons 2005; Arnquist and Rowe 2005). Nonetheless, polyandry is a taxonomically widespread phenomenon, also common in insects and mites (e.g. Dickinson 1997; Witaliński 1999; Simmons 2001; Hosken and Stockley 2003). Both genetic and non-genetic benefits can promote the evolution of polyandry, e.g. fertilization by high quality sperm due to sperm competition, increased genetic diversity among offspring, reduced risk of inbreeding and genetic incompatibility; sperm replenishment, increased female fertility and fecundity due to male-donated nutrients and ejaculate defensive compounds, increased parental care and avoidance of infanticide and predation (rev. Simmons 2001, 2005; Hosken and Stockley 2003).

Eriophyoid mites (Acari: Eriophyoidea) are a group of minute (ca. 0.1–0.3 mm long), four-legged and highly specialized herbivores forming galls or living freely on plants. Many species are economically important, causing rusting or various distortions of growth of plant tissue, and transmitting plant viruses (Lindquist et al. 1996). Eriophyoids transfer sperm indirectly, via spermatophores deposited on a substrate and without pair formations between males and females (so called sex dissociation or non-pairing) (Thomas and Zeh 1984; Oldfield and Michalska 1996; Proctor 1998). Spermatophores of eriophyoids consist of a head with a central sperm reservoir, a stalk and a “foot” by which the spermatophore is attached to the substrate. Up to date behavioural observations on the visitations of spermatophores by eriophyoid females were conducted in *Aculus fockeui* (Nalepa and Trouessart), *A. schlechtendali* (Nalepa), *Epitrimerus pyri* (Nalepa) and *Phyllocoptruta oleivora* (Ashmed) (Oldfield et al. 1972; Oldfield and Newell 1973; Oldfield 1988). When mounting a spermatophore, females of various species behave similarly (Oldfield and Michalska 1996). First, they sway from side to side a few times on a spermatophore and then they become motionless for several seconds, lying on a spermatophore head at the region of the genital opening (which is situated ventrally just behind the coxae of the second pair of legs) while their cauda rested on the leaf surface. Finally, they resume gross movements and slightly pull the spermatophore, which remains attached to the leaf surface when the females leaves. Behavioural and microscopic investigations by Oldfield et al. (1972) and Oldfield and Newell (1973) revealed that females of *A. fockeui* pick up sperm from only a single spermatophore in their lifetime and deposit sperm in either the left or right spermatheca only. In virgins of *A. fockeui*, both spermathecae are similar in size and ellipsoidal. However, after the spermatophore visitation, the sac with sperm becomes larger and spheroid. By contrast, in other eriophyoids, the sperm-filled spermatheca can be turgid but ellipsoidal (Oldfield 1973). The size of the sperm reservoir of the *A. fockeui* spermatophore corresponds to the size of the sperm-filled spermatheca (Oldfield et al. 1972). A similar relationship between sperm reservoir and sperm-filled spermatheca as well as asymmetrical sperm storage, was reported in many other eriophyoid species (34 species in total), mostly from dicotyledonous host plants. By contrast, symmetrical sperm storage, in which sperm is stored in both spermatheca, prevailed in eriophyoid species that associated with gymnosperm and monocot host plants (Oldfield 1973, 1999). In three examined species, *Aceria cynondonis* (Wilson) (previously *Eriophyes cynondonis*), *Aceria tulipae* Keifer (previously *Eriophyes tulipae*) and *Trisetacus ehamnni* Keifer the sperm-filled spermathecae were as large or larger than the sperm reservoir of a spermatophore, which suggested that females with symmetrical sperm storage pick up sperm multiply (Oldfield 1973). However, no behavioural studies confirmed polyandry in these eriophyoids.

In eriophyoids, monandry may be advantageous, because soon after insemination from a spermatophore females can migrate to new, more profitable patches and reproduce without wasting time and energy on finding subsequent spermatophores. However, by picking up sperm from only a single spermatophore the eriophyoid females risk becoming inseminated for their whole lifetime with sperm of low quality and quantity. Under such conditions, female reproductive success would substantially decrease and repeated visitations of spermatophores, as well as polyandry, might be promoted.

The aim of this study is to examine spermatophore visitation behaviour and symmetry of sperm storage in two free-living species: *Cecidophyopsis hendersoni* (Keifer) from *Yucca* sp. and *A. allotrichus* (Nalepa) (syn. *Vasates robiniae* Nalepa) from the black locust tree *Robinia pseudoaccacia* L. This would confirm monandry or polyandry in those species. In *C. hendersoni,* the effect of the presence of conspecifics and leaf condition on spermatophore deposition rate has been previously studied (Michalska and Shi 2004). On non-infested leaves, males deposit considerably fewer spermatophores than on infested leaves from which conspecifics were washed off. When non-infested leaves are offered, males decrease their spermatophore deposition rate on those leaves which are older or mechanically damaged. Virgin females have a stimulating effect on spermatophore deposition rate only on leaves previously infested by conspecifics, in this mite. As *C. hendersoni* inhabits a monocotyledonous host plant, I suspect that females of this species might pick up sperm from spermatophores multiply in their lifetime. By contrast, females of *A. allotrichus* appear to be monandrous. Firstly, this eriophyoid stores sperm asymmetrically (Oldfield 1999). Secondly, males deposit spermatophores and guard females on their emergence sites (Michalska 1999). Guarding of pre-emergent females strongly suggests that females become inseminated once or only a few times during their lifespan, shortly after emergence (Thornhill and Alcock 1983). On the emergence sites, *A. allotrichus* females visit spermatophores as soon as their genital coverflaps are free and most of their old exuvia are shed after moulting. When they are not surrounded with spermatophores they return to the emergence site several times searching for spermatophores and making greater and greater circles until they finally wandered off (Oldfield and Michalska 1996). In this paper, I examined the behaviour of *A. allotrichus* and *C. hendersoni* females in relation to spermatophores during the whole female lifespan, from the day of emergence to the death of the female. I also examined spermathecae in squashed females right after the visitation of spermatophores as well in females randomly taken from the population to confirm asymmetrical or symmetrical sperm storage in both species.

## Materials and methods

The original population of *C. hendersoni* was sampled from infested yucca (*Yucca* sp.) plants grown in a glasshouse in Brzeg Dolny (Poland). The eriophyoids used in the experiments were obtained from potted yucca (*Yucca* sp.) plants grown in a climatic room at 26 °C and 16/8 L/D photoperiod. *Aculops allotrichus* was collected from black locust trees (*R. pseudoaccacia*) grown on the campus of Warsaw University of Life Science. Apart from the leaf-lets populated with *A. allotrichus* I also collected “clean” leaf-lets from the black locust trees that were non-infested by *A. allotrichus*. To keep the sampled leaves of yucca or the leaflets of black locust tree in turgor, they were put into plastic boxes lined with wet cotton. As soon as leaves were brought to the laboratory, detached leaf cages (for details see Michalska and Shi 2004; Michalska 2000, 2005) were constructed and the eriophyoids chosen for a test were transferred to them. To construct cages for *A. allotrichus*, ‘clean’ leaf-lets of black locust were used. In *C. hendersoni,* males deposited many more spermatophores and females laid many more eggs on the infested yucca leaves from which eriophyoids were washed off than they did on the non-infested leaves (Michalska and Shi 2004; Michalska, pers. obs.). Therefore, in this study, only the ‘washed off’ infested leaves of yucca were applied.

To estimate whether the eriophyoids pick up sperm from one or more spermatophores in their lifetime, females of each species were exposed to the presence of spermatophores from the day of their emergence until the end of their lives. Twelve *C. hendersoni* females were observed every day, for 12 days. *Aculops allotrichus* females (N = 25) were exposed to spermatophores on the day of emergence (1st day) and then every other day, on 3rd, 5th, 7th, 9th, 13th and 15th day, until the death of the females. *Aculops allotrichus* was observed at a lower frequency than *C. hendersoni*, as previous observations on asymmetrical sperm storage and spermatophore visitation by *A. fockeui* (Oldfield and Newell 1973), as well as asymmetrical sperm storage (Oldfield 1999) and guarding behaviour in female emergence sites of *A. allotrichus* (Michalska 1999), all suggested that *A. allotrichus* females should pick up sperm from single spermatophores in their lives and mostly on the day of emergence.

In both species, females were tested in the experimental cages, on the leaf arenas of 5.5 mm in diameter (Michalska 2000; Michalska and Shi 2004), each with an aggregation of 8–10 fresh spermatophores. The spermatophore aggregations were formed by groups of 6–8 ‘random’ males that were collected from the infested leaves of yucca plants or black locust trees. Each male group deposited spermatophores only on one leaf arena. The number of spermatophores each male allocated to the spermatophore aggregation was not controlled. Males deposited spermatophores under light conditions for 6 h. After that time, males were removed and the experiments started.

To obtain females of *C. hendersoni* and *A. allotrichus* the quiescent female nymphs were collected from infested leaves of the appropriate host plant and then placed singly into the ‘rearing’ cages (in which females spent their whole life), onto leaf arenas 6.5 mm in diameter (Michalska 2000; Michalska and Shi 2004). In both species, female quiescent nymphs are bigger than male quiescent nymphs and therefore they can be easily distinguished from each other. The ethological observations were carried out the next day, in the early afternoon, after all the females had emerged. The females were then transferred into experimental cages, placed in the centre of leaf arenas and observed for the next half hour. At each observation, the number and time of spermatophore visitations were recorded. For 11 spermatophore visitations by *C. hendersoni* and 7 visitations of spermatophores by *A. allotrichus* the time of mounting a spermatophore, lying on a spermatophore head and dismounting was additionally estimated. The time was measured using a stopwatch. The time was taken to the nearest second and rounded up. The observations were made at 50–100× magnification under a dissecting microscope fitted with a cold light source Olympus Highlight 3100 (equipped with a halogen reflector lamp; colour temperature: 3,090 K). After each observation was completed, the females were transferred back to the “rearing” cages with new, fresh leaves where the females were maintained until the next observations. Every day, the number of eggs laid in the cages was additionally counted. Mites were manipulated using an eye-lash glued onto the tip of a wooden stick. Mites and cages were kept in plastic trays lined with wet cotton, in a Sanyo growth chamber at 26 °C, 85–89 % RH and 16/8 L/D photoperiod.

I examined spermathecae for the presence of sperm in ten females of *C. hendersoni* and *A. allotrichus*, which had been randomly chosen from a mite population, and in four females of *C. hendersoni* and five females of *A. allotrichus,* which had been exposed to spermatophores just after emergence. Newly emergent females were obtained from quiescent female nymphs and released to the experimental cages with 8–10 spermatophores, following the methods I have described above. The females of *C. hendersoni* were inspected only after a half-hour-long observation, during which the females visited 2–3 spermatophores. By contrast, *A. allotrichus* females were examined immediately after the visitation of just one spermatophore. To examine spermathecae for the presence of sperm, the Oldfield et al. (1972) method was applied. Both, ‘random’ and newly inseminated females were put singly into a drop of saline solution (0.9 % NaCl), in a dorso-ventral position. Their caudal part was then cut off using a needle and the acaudal part was covered by the coverslip. Coverslips were gently pressed so most of the body content was extruded through the place of the amputated rear body end. Spermathecae were then inspected for the presence of non-motile, ellipsoidal sperm cells ca. 2 μm in diam with a relatively large, dark nucleus. Similar sperm cells have been observed in a drop of saline solution, into which spermatophores of both species were placed and to which sperm from spermatophores was released (Michalska 2011; Michalska K., unpubl.). Spermathecae were examined under a phase-contrast dissecting microscope (Olympus CX41) fitted with a Colour View III CCD camera and the imaging software for life science microscopy, Cell^B^ (Olympus Soft Imaging System).

Statistical analysis was performed using Statgraphics Plus 4.1. In *A. allotrichus*, I compared the time of visitation of the first and the second spermatophore using paired *t* test, and the time of visitation of the first out of two spermatophores and the single spermatophore using a two-sample *t* test. Both tests were applied at significance level ∞ = 0.05. Before tests the data were examined for normality and homogeneity of variance (Sokal and Rohlf 1995). Data were shown as mean ± SE.

## Results

### Behaviour of *Cecidophyopsis hendersoni* and *Aculops allotrichus* females during visitation of spermatophores

In both species, most females changed their behaviour when they approached a spermatophore. At the distance of approximately one length of a female body from the spermatophore aggregation, females either stopped or markedly slowed down, approaching spermatophores on ‘stiff legs’. Such behaviour was exhibited by 8 out of 9 females of *C. hendersoni* prior to the visitation of 18 out of 23 spermatophores and by 15 out of 17 females of *A. allotrichus* prior to the visitation of single spermatophores. For the rest of the tested females, one female of *C. hendersoni*, prior to the visitation of three spermatophores and five *C. hendersoni* females, prior to the visitation of one spermatophore, as well as two females *A. allotrichus* that visited single spermatophores, slowed down their walking only when they entered the spermatophore aggregation. Within the aggregation females began to search for the ‘proper spermatophore’, turning their bodies alternately to the left and right. Females of both, *C. hendersoni* and *A. allotrichus* examined spermatophores in two ways: (1) they tapped the spermatophore head with the first pair of legs and/or touched the sperm reservoir using their tarsal claws or (2) they crouched down, while walking over a spermatophore, and rubbed up against it using their genital cover-flaps.

**Fig. 1:**
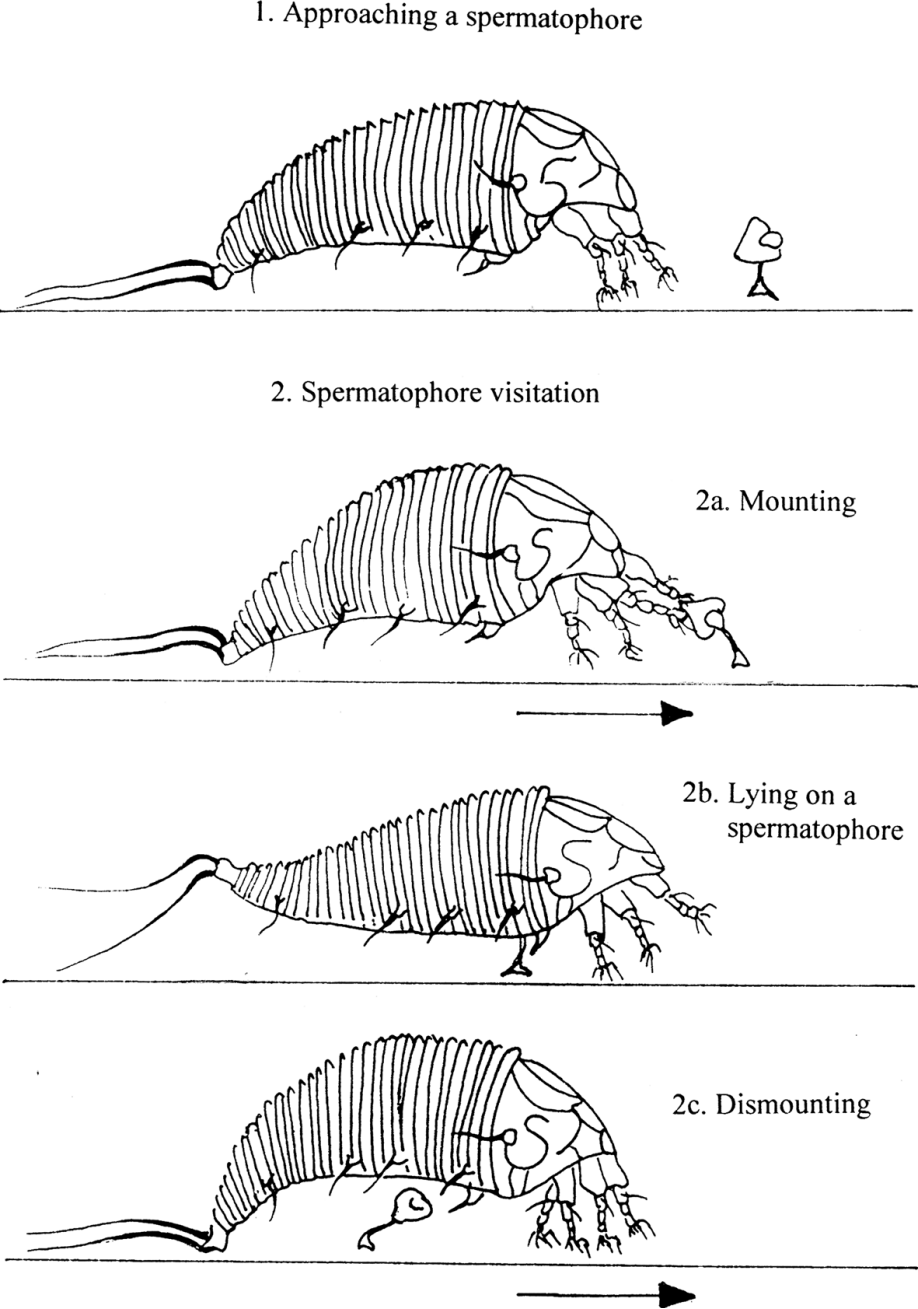
Phases of spermatophore visitation by an eriophyoid female. An *arrow* indicates the direction of female movement

In both species, the visitation of a spermatophore consisted of three phases: (1) mounting, (2) lying on the spermatophore head, and (3) dismounting (Fig. 1). When mounting a spermatophore, females brought a spermatophore closer to them with their front legs, and keeping it in place they moved forward over it. Then the females swung their bodies from side to side, as if they tried to fit their genital opening with the sperm reservoir placed within the spermatophore head. In *C. hendersoni*, the mounting phase lasted on average 11.45 ± 3.19 s (N = 11; min–max: 3–35) and in *A. allotrichus*, 10.5 ± 3.19 s (N = 7; min–max: 4–20). Finally, the females of both species became motionless on the spermatophore head with their legs hanging loosely (Fig. 1). In this phase some females waved their legs from time to time. *C. hendersoni* females spent on average 39.09 ± 7.86 s (N = 11; min–max: 3–96) lying on spermatophores while *A. allotrichus* on average 115.38 ± 35.95 s (N = 7; min–max: 12–228) s. Dismounting the spermatophore took a female of *C. hendersoni* on average 4.55 ± 0.41 s (N = 11; min–max: 3–7) and *A. allotrichus,* 5.26 ± 0.49 s (N = 7; min–max: 3–7). First, the female swung her body vigorously from side to side and then she moved forward slowly until a spermatophore sprang off, returning to a perpendicular position on the leaf (Fig. 1). After dismounting, females of both species usually stopped in the close vicinity of the spermatophore and began feeding. However, some females could also exhibit a sort of excitation with the ‘used’ spermatophore. Before feeding they returned to the spermatophore and walked over it several times, rubbing up against it vigorously. Also, after feeding females of *C. hendersoni* and *A. allotrichus* did not abandon the spermatophore aggregation immediately. They returned to the aggregation several times, making greater and greater circles until they finally went away. The females that no longer showed interest in spermatophores walked through the aggregation fast, without stopping and examining the spermatophores.

### Time and frequency of spermatophore visitations by *Cecidophyopsis hendersoni*

**Fig. 2:**
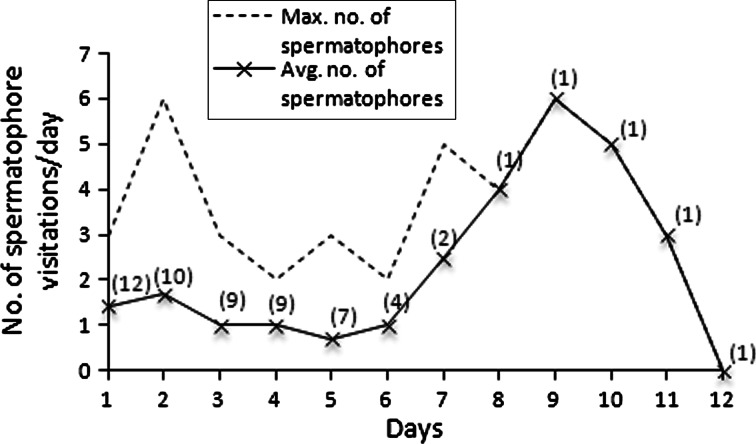
The average and maximum number of spermatophores visited by *Cecidophyopsis hendersoni* females during the successive days of their lifetimes. In *brackets* number of females that were observed each day

Females of *C. hendersoni* lived on average 5.67 ± 0.85 days (N = 12; min–max: 2–12). One out of the twelve tested females was not interested in spermatophores and died on the second day of her life. Females visited spermatophores multiply, mostly day after day, on average 1.54 ± 0.3 spermatophores per day (N = 12; min–max: 0–6) (Fig. 2). The visitation of the spermatophore lasted on average 51.69 ± 2.38 s (N = 97; min–max: 10–120). Four females started to visit spermatophores as soon as the first day, and seven other females on the second day after emergence. Only one virgin female mounted the first encountered spermatophore. The other females usually examined all spermatophores in an aggregation before the final visitation of the preferred spermatophores. In two females, a sort of excitation about three used spermatophores was also observed.

Five out of twelve females laid eggs, on average 0.12 ± 0.05 eggs per day, 1–3 eggs during their whole lifetime. Egg lying started at its earliest on the 3rd day after female emergence. The female that lived for the longest period of time (12 days), visited spermatophores every day except the first, seventh and twelfth days. She laid three eggs during her life and performed visitations of 33 spermatophores.

### Time and frequency of spermatophore visitations by *Aculops allotrichus*

*Aculops allotrichus* females lived on average 5.84 ± 0.79 days (N = 25; min–max: 1–15). Only one female did not show any interest in spermatophores, and she died on the second day after emergence. 15 females visited spermatophores on the first day after emergence, 6 females did so on the third day and 3 females visited spermatophores only on the fifth day after emergence.

Five out of 24 females mounted the spermatophore that they first approached. Others, before mounting a spermatophore, examined a few or all the other spermatophores in the aggregation. For 4 of the 5 females that visited the first approached spermatophore, this spermatophore was also the only one they mounted in their lifetime, while for the fifth female it was one of the two spermatophores she visited in her lifetime.

In total, 16 out of 24 females visited only one spermatophore in their lifetime (they were not interested in spermatophores in the days following their exposure to spermatophores), while 8 females, which is one-third of all those tested, visited two spermatophores in their lives. The mean time of spermatophore visitations was 115.74 ± 14.51 s (N = 31; min–max: 20–315).

When females visited two spermatophores in their lifetime, either they mounted them both on the same day during the half hour exposure to spermatophores (six females), or the visitation of the second spermatophore occurred at the other exposure day (two females), at an interval of 1–6 days. Visitation of the first and the second spermatophore consisted of similar phases of mounting, lying on a spermatophore and dismounting. Also, the time of visitations of the first and the second spermatophore was similar (paired-sample *t* test: N = 8; *t* = −1.15371; *P* = 0.29) (Table 1). Moreover, the time of the visitation of the first out of two spermatophores did not differ significantly from that spent on the visitation of the single spermatophore in a female lifetime (two-sample *t* test: N_1_ = 15; N_2_ = 8; *t* = 1.31292; *P* = 0.20) (Table 1). A similar result was obtained when four cases of females, which visited a single spermatophore on the first day of the exposure to spermatophores but died before the second exposure day, at which they theoretically could have visited the second spermatophore, were excluded from the analysis (two-sample *t* test: N_1_ = 11; N_2_ = 8; *t* = 1.32172; *P* = 0.20).

**Table 1:** The times of single and double visitations of spermatophores by females of *Aculops allotrichus*

	Single visitation of spermatophores	Double visitation of spermatophores	
		1st spermatophore	2nd spermatophore
Mean ± SE time (s)	129.0 ± 23.27	83.75 ± 17.13	122.88 ± 31.34
Minimum and maximum time (s)	20–315	35–170	25–280
Number of observations	15	8	8

Ten out of 24 females laid eggs during the experiment. One female laid an egg on the first day while the others, at the earliest, on the 4th day after emergence. They lived for six or more days and oviposited at the rate, on average, of 0.25 ± 0.03 eggs per day, from one to six eggs during their whole lifetime. Nine females laid eggs only after they visited a spermatophore, while one female already deposited an egg before spermatophore visitation.

### Examination of spermathecae for the presence of sperm

**Fig. 3:**
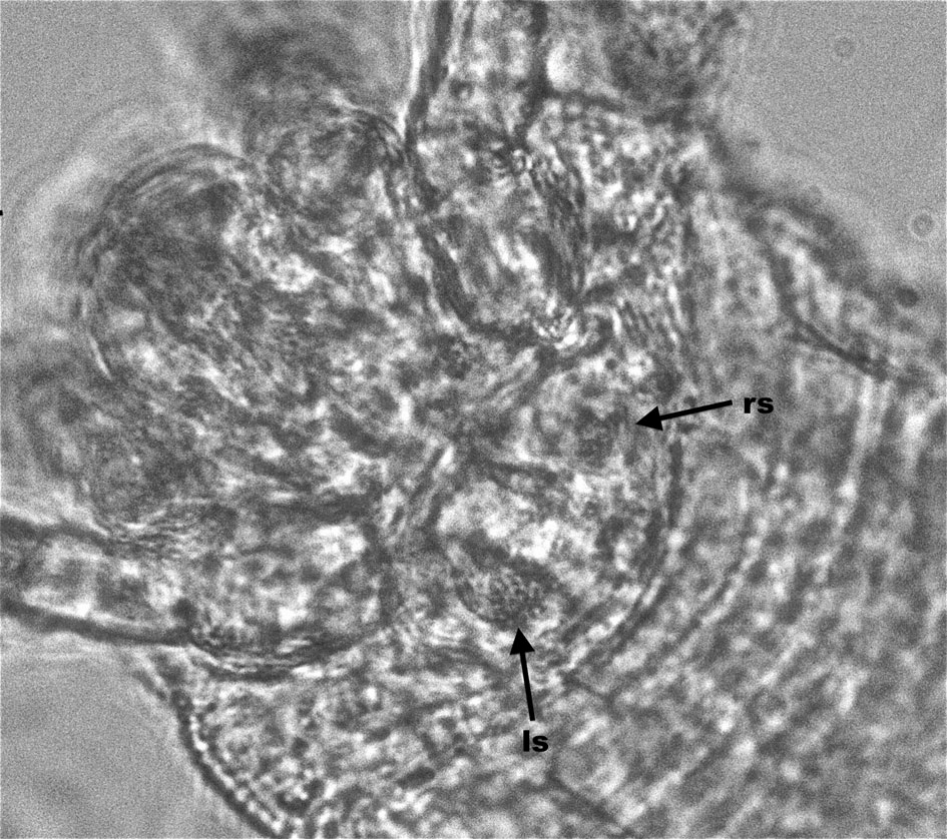
Photomicrograph of the paired spermatecae and symmetrical sperm storage in *Cecidophyopsis hendersoni*. Both *left* (ls) and *right* (rs) spermatheca filled with spermatozoa

**Fig. 4:**
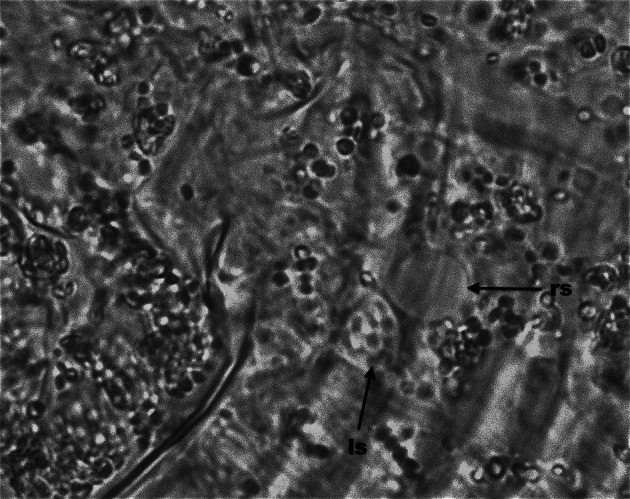
Photomicrograph of the paired spermatecae and asymmetrical sperm storage in *Aculops allotrichus*. One spermatheca (ls) is filled with sperm and the other spermatheca (rs) is empty

In all females of *C. hendersoni*, either randomly chosen from a population (N = 10) or inspected just after the visitation of 2–3 spermatophores (N = 4 females), sperm were present in both spermathecae (Fig. 3). By contrast, all *A. allotrichus* females, regardless of whether they were ‘random’ (N = 10) or just after the visitation of a single spermatophore (N = 5), had spermatozoa only in one, the left or right, sac while the other sac remained empty (Fig. 4). In both species, spermathecae were spheroid. The spermathecae of newly inseminated virgins were filled with sperm, while those of ‘random’ females were filled with sperm to a varying degree.

## Discussion

In this study, visitations of spermatophores by *A. allotrichus* and *C. hendersoni* consisted of similar phases of mounting, lying on a spermatophore head and dismounting, which were also observed in other eriophyoid species i.e. *A. fockeui*, *A. schlechtendali*, *E. pyri* or *P. oleivora* (Oldfield and Newell 1973; Oldfield 1988; Oldfield and Michalska 1996). However, while *C. hendersoni* females were polyandrous and visited spermatophores almost every day, from the day of emergence until death, in *A. allotrichus*, the majority of females visited spermatophores once, and the others at most twice in their life.

Previous investigations (Oldfield 1973) suggested that eriophyoid species storing sperm symmetrically should become inseminated from two or more spermatophores in their lives. This relationship has been confirmed for the first time in this study. The females of *C. hendersoni* visited several spermatophores in their lifetimes and those examined just after the visitation of 2–3 spermatophores, stored sperm in both spermathecae. Moreover, all females randomly collected from a population exhibited symmetrical sperm storage.

By contrast, *A. allotrichus* females deposit spermatozoa only in one spermathecae (Oldfield 1999), as was also revealed in the microscopic and behavioural observations in this study. However, nearly one-third of females visited two spermatophores, instead of one, in their lives. Both the visitation of the first and the second spermatophore consisted of similar phases of mounting, lying on a spermatophore and dismounting; moreover, the time spent on the visitation of the second spermatophore did not differ significantly from that of the first spermatophore and was also similar to the visitation time of a single spermatophore in a female life. This suggests that *A. allotrichus* females may pick up sperm from two spermatophores.

In this study, I examined the spermathecae of *A. allotrichus* females that visited just one, not two, spermatophores in their lives, and they all stored sperm asymmetrically. Moreover, all females I collected randomly from a population, among which some individuals might have picked up sperm from two spermatophores, also exhibited asymmetrical sperm storage. This is in disagreement with the previous findings on *A. fockeui* and other eriophyoids (Oldfield et al. 1972; Oldfield and Newell 1973; Oldfield 1973; Oldfield and Michalska 1996) suggesting that species with asymmetrical sperm storage should become inseminated from only one spermatophore in their lifespan.

In the study by Oldfield and Newell (1973), all ten virgin *A. fockeui* females promptly visited and become inseminated by single spermatophores, but they failed to visit any fresh spermatophores in the following trials (on the 4, 8, 12 and 16th days of their adult lives), even when they became old and started to produce male offspring only (which indicates that sperm stores have already run out in these haplodiploids). In the other study (Oldfield et al. 1972), however, some newly inseminated females mounted a second spermatophore, but for a few seconds only. Oldfield (personal communication) argued that such mountings lasted too short a time to facilitate sperm transfer. Also in this study, in *A. allotrichus* and *C. hendersoni,* short, only 10–20 s long visitations of spermatophores, were recorded. It is likely that during mounting the females conclusively examined the quality of a spermatophore and abandoned it quickly (without insemination or picking up only some portion of sperm) when it turned out to be of lower quality or inappropriate. One cannot exclude, however, the possibility that some females simply failed to transfer sperm properly from the spermatophore. Such a case was described in the sex dissociated springtail *Orchesella cincta* (L.), in which females between moultings mostly pick up sperm from a single spermtophore (Gols et al. 2004). Paternity analysis of the offspring produced by the ‘double mated’ female revealed that only the visitation of the second spermatophore had been successful. The authors did not exclude, however, the possibility that the female collected sperm from two spermatophores deposited by the same male, in which case the double insemination could not have been detected. It must be stressed that in eriophyoids, quick visitation of a spermatophore may be also a consequence of female excitement after insemination and may lead to the prolonged ‘molesting’ of some used spermatophores observed in this study.

In many species of insects and mites, females that were previously considered to mate only once in their lifetime turned out to re-mate with a certain frequency (Helle 1967; rev. Eberhard 1985; Potter and Wrench 1978; Ridley 1988; Khanh et al. 2005; Burton-Chellew et al. 2007). Benefits arising from re-mating and polyandry, e.g. sperm replenishment, nutrient donation, genetic diversity of offspring, sperm competition and ‘good genes,’ as described for copulating females (rev. Hosken and Stockley 2003), might be similar for females from sex dissociated taxa. For example, the number of spermatozoa per spermatophore varies greatly among *A. allotrichus* males (Michalska 2011). Thus, *A. allotrichus* females could have visited the second spermatophore if they did not obtain enough sperm from the first spermatophore to fertilize their eggs. Eriophyoid females may also accomplish double or multiple insemination to increase the genetic diversity of their offspring (and so avoid the risk that all of their offspring will be fathered by a male of bad quality). However, repeated visitations of spermatophores can also incur some cost of time and energy that the female has to devote to seeking fresh spermatophores. *Cecidophyopsis hendersoni* form very dense, multi-generational populations on evergreen yucca leaves (Michalska and Shi 2004; Michalska K., pers. obs.), in which the chances that a female will encounter a fresh spermatophore appears to be relatively high. Thus, she can visit spermatophores every day and even several times per day. By contrast, *A. allotrichus* lives in a much lower density; leaves of the black locust tree quickly become rusted by mite feeding and females have to seek new young leaves for lying eggs. In contrast to females, males usually stay on older leaves until the quiescent female nymphs are present and deposit spermatophores beside them (Michalska K., pers. obs.). Thus, females would wait several days on the young leaves for new generation males and their spermatophores. However, when an *A. allotrichus* female emerges she is usually surrounded with spermatophores of various males (as a result of joint-guarding) (Michalska et al. 2010), so the female can accomplish double insemination without the wastage of time and energy involved in searching for spermatophores. Indeed, as this study revealed, in *A. allotrichus*, the majority of re-mating females visited the second spermatophore on the same emergence day, within the short time of a 30 min-long observation. It is likely that the receptivity of *A. allotrichus* females depends on the amount of secretions contained in spermatophores, and if they do not receive enough of those substances or if their amount in a female spermatheca declines (e.g. with female aging), females may re-mate. Such a phenomenon was observed in the Mediterranean fruit fly *Ceratitis capitata* (Chapman et al. 1988; Mossinson and Yuval 2003). In this species, females that chose to re-mate possessed fewer sperm in their spermatheca than those that did not choose to re-mate (Mossinson and Yuval 2003). Interestingly, nearly 50 % of females re-mated soon (on the 2nd day) after the first mating while only 0–10 % on the 12th day (Chapman et al. 1988).

This study also showed that females of both species as a rule examined several spermatophores before the mounting of one. It is likely that eriophyoid females need some stimulation from the other spermatophores before mounting the spermatophore and picking up sperm from it. However, one cannot also exclude the possibility that the eriophyoid females simply chose among the spermatophores and mounted that preferred one. It must be stressed that receptive females of the other eriophyoid, *A. fockeui* (Oldfield et al. 1970) can distinguish between old and fresh spermatophores and do not pick up sperm from spermatophores that are more than 3 days old. Thus, in future work, preference tests should be done to determine whether eriophyoid females are choosy about spermatophores and whether their choosiness is based on the different genetic quality of spermatophores or, perhaps, the various ages of spermatophores they encounter. Non-pairing females may recognise male ‘good genes’ on the basis of some spermatophore traits, especially their pheromones, as well as other substances within a sperm drop (Proctor 1998; Zizzari et al. 2009). In the eriophyoid spermatophores, the presence of pheromones has not yet been confirmed, although some behavioural observations, e.g. female response toward spermatophores from a distance, as well as discrimination between old and new spermatophores, strongly suggest it (Michalska et al. 2010). Female choice has been demonstrated in the sex dissociated springtail *O. cincta* (Hedlund et al. 1990; Gols et al. 2004; Zizzari et al. 2009). Females chose spermatophores of a particular male and through this choice they gained more fertile male offspring. In future work on *A. allotrichus* and *C. hendersoni*, paternity analysis of female offspring should be carried out to confirm that the females which visited two or more spermatophores did indeed become inseminated by sperm from those males. In this context, the mechanism of sperm competition could also have been determined.

In insects and mites, sperm from different males mix randomly in spermatheca, or there is sperm precedence of the first or, most frequently, the last male (Simmons 2001). Also, the reproductive success of a non-pairing male may depend on the number of sperm per spermatophore or the sequence of visitations of his spermatophores in relation to the spermatophores of competitor males. Moreover, the effectiveness of mating may also rely on the interval between the first and the second insemination. In *Tetranychus urticae* (Helle 1967) or *T. kanzawai* (Oku 2008), for example, the first copulation is effective. First male sperm precedence appears to be adaptive in this case, since, similarly to *A. allotrichus*, tetranychid males spend time and energy on the prolonged guarding of pre-emergent females. However, when the first copulation by *T. urticae* males was interrupted or the time between the first and the second copulation was shorter than 24 h, the second insemination was effective in some cases (Potter and Wrench 1978; Satoh et al. 2001). Similarly, one cannot exclude the possibility of some differences in the effectiveness of the repeated inseminations in *A. allotrichus* between females that accomplish the second insemination just after the visitation of the first spermatophore and those that visited the second spermatophore much later in their lifetimes.

The volumetric comparison of the sperm reservoir and spermatheca in three species of eriophyoids *A. cynondonis*, *A. tulipae* and *T. ehamnni* that store sperm symmetrically revealed that each spermatheca may be filled with sperm from 1 to 2 spermatophores (Oldfield 1973). In this study, *C. hendersoni* females clearly picked up sperm from many more spermatophores that seem to be required for egg fertilization. They laid a maximum of three eggs and visited up to 33 spermatophores in their lifetime, a maximum of 6 spermatophores at one observation. The females that did not lay eggs also visited up to 7 spermatophores in their lifetime. Thus, one cannot exclude the possibility that in this species females systematically remove or digest the old sperm and refill their spermatheca with new sperm supplies. Numerical and functional elimination of sperm by females at mating is a widespread phenomenon among insect species in which females and males copulate (Eberhard 1996; Dickinson 1997; Showalter 2006; Pai and Bernasconi 2007). Females can digest or extrude sperm after copulation, block sperm transfer into spermatheca, reduce sperm viability via the hostile environment of their reproductive tract etc. Females can benefit from sperm removal by promoting sperm competition, manipulating paternity, obtaining nutrition, eliminating sperm that bear mutations, etc. (rev. Bernasconi et al. 2002). Thus, in future studies, paternity analysis is required to confirm multiple inseminations by *C. hendersoni* females and the possible mechanism of sperm elimination in this species.
